# Chromatin complexes subunit BAP18 promotes triple-negative breast cancer progression through transcriptional activation of oncogene S100A9

**DOI:** 10.1038/s41419-022-04785-x

**Published:** 2022-04-28

**Authors:** Yin-Ling Zhang, Ling Deng, Li Liao, Shao-Ying Yang, Shu-Yuan Hu, Yan Ning, Fang-Lin Zhang, Da-Qiang Li

**Affiliations:** 1 Fudan University Shanghai Cancer Center and Institutes of Biomedical Sciences, Fudan University, Shanghai, 200032 China; 2 Cancer Institute, Shanghai Medical College, Fudan University, Shanghai, 200032 China; 3 Department of Pathology, Obstetrics and Gynecology Hospital, Fudan University, Shanghai, 200032 China; 4 Department of Breast Surgery, Shanghai Medical College, Fudan University, Shanghai, 200032 China; 5 Shanghai Key Laboratory of Breast Cancer, Shanghai Medical College, Fudan University, Shanghai, 200032 China; 6 Shanghai Key Laboratory of Radiation Oncology, Shanghai Medical College, Fudan University, Shanghai, 200032 China

**Keywords:** Breast cancer, Oncogenes

## Abstract

Triple-negative breast cancer (TNBC) is a highly lethal disease due to aggressive clinical phenotype and the lack of validated therapeutic targets. Our recent quantitative proteomic analysis of 90 cases of TNBC tissues and 72 cases of matched adjacent normal tissues revealed that the expression levels of BPTF-associated protein of 18 KDa (BAP18), a component of the MLL1 and NURF chromatin complexes, were upregulated in TNBC tissues relative to normal tissues. However, the biological function and the underlying mechanism of BAP18 in TNBC progression remain unexplored. Here, we report that BAP18 promoted TNBC cell proliferation, migration, and invasion in vitro and xenograft tumor growth and lung colonization in vivo. Mechanistic investigations revealed that S100 calcium-binding protein A9 (S100A9), a member of the S100 protein family that is frequently upregulated in breast tumors and acts as an oncogenic driver in breast cancer progression, was a downstream target gene of BAP18. BAP18 was recruited to histone H3 trimethylation at lysine 4 (H3K4me3)-marked promoter of S100A9 and enhanced its promoter activities. Notably, knockdown of BAP18 by short hairpin RNA in TNBC cells suppressed xenograft tumor growth in mice, the noted effect was partially reverted by re-expression of S100A9 in BAP18-depleted cells. Taken together, these results suggest that BAP18 promotes TNBC progression through, at least in part, transcriptional activation of oncogene S100A9, and represents a potential therapeutic target for TNBC.

## Introduction

Breast cancer is the most commonly diagnosed cancer among females with high heterogeneity in clinicopathological features and molecular characteristics [1, 2]. According to the expression status of estrogen receptor (ER), progesterone receptor (PR), and human epidermal growth factor receptor 2 (HER2), breast cancer is subclassified into three main molecular subtypes, including hormone receptor-positive, HER2-positive, and triple-negative breast cancer (TNBC) [3]. TNBC is defined by the lack of ER, PR, and HER2 expression and accounts for about 15–20% of all breast cancers [4]. In contrast to other molecular subtypes, TNBC occurs most frequently in young women, exhibits highly aggressive and metastatic phenotypes, and lacks validated therapeutic targets, thus resulting in a worse clinical outcome [4–6]. To date, clinical management of TNBC is still challenging due to poor understanding on its molecular etiology.

Emerging evidence shows that epigenetic regulators play a fundamental role in TNBC pathogenesis and represent promising therapeutic targets [7–11]. BPTF-associated protein of 18 KDa (BAP18) is a poorly characterized protein, encoded by chromosome 17 open reading frame 49 (C17orf49) gene. Of note, BAP18 contains a SWI3, ADA2, N-CoR and TFIIIB (SANT) domain, which is usually present in the subunits of chromatin-remodeling complexes and has a central role in chromatin remodeling by functioning as a histone interacting module [12–14]. Indeed, BAP18 has been identified as a component of the mixed lineage leukemia protein 1 (MLL1) and nucleosome remodeling factor (NURF) chromatin complexes and as a reader for histone H3 trimethylation at lysine 4 (H3K4me3) [15, 16]. In addition, BAP18 directly interacts with DPY-30, a subunit of mammalian COMPASS-like complex that regulates global H3K4me3 levels [17]. These observations indicate a potential role for BAP18 in transcriptional regulation. In support of this notion, BAP18 has been shown to interact with androgen receptor (AR) and to enhance AR-mediated transcription in granulosa cells [18]. Consequently, reduced BAP18 expression impairs granulosa cell function, which may be implicated in polycystic ovary syndrome pathophysiology [18]. In addition, BAP18 facilitates the recruitment of MLL1 subcomplex to the promoters of AR target genes to coactivate AR-mediated transcription in prostate cancer cells, thus promoting prostate cancer growth [19]. Similarly, BAP18 recruits the core subunits of COMPASS-like complex to the promoter regions of estrogen receptor α (ERα) to enhance ERα-mediated transactivation [20]. As a result, the expression levels of BAP18 are associated with the sensitivity of ERα-positive breast cancer cells to endocrinal therapy [20]. In addition, BAP18 promotes the growth of oral squamous cell carcinoma by upregulation of cyclin D1/2 [21]. Despite these advances, the biological function and related mechanism of BAP18 still remain largely unknown.

S100 calcium-binding protein A (S100A) family of proteins comprises over 20 members, and most of them are frequently dysregulated in human malignancies including breast cancer [22]. A case in point is S100 calcium-binding protein A9 (S100A9), which is strongly upregulated in ductal carcinoma in situ of the breast [23] and overexpressed in poorly differentiated invasive breast ductal carcinoma [24], especially in the basal-like and HER2-amplified breast tumors [25]. Moreover, increased S100A9 expression is associated with poor tumor differentiation [24], poor pathological parameters [26], high tumor grade [27], increased tumor recurrence and metastasis [28, 29], and a poor prognosis of patients with breast cancer [25, 30, 31]. As a secretory cytokine, S100A9 accelerates breast cancer growth and metastasis upon binding to a cell surface receptor, melanoma cell adhesion molecule (MCAM) [32]. The S100A9-MCAM axis activates ETS translocation variant 4 (ETV4), which in turn transcriptionally upregulates ZEB1 to induce epithelial-mesenchymal transition and metastasis of breast cancer cells [32]. In support of these observations, S100A9 also contributes to H-ras-mediated human breast epithelial cell migration and invasion [33] and to the recurrence of breast tumors with chromosome 1q21.3 amplification [28]. In addition, the expression levels of S100A9 are associated with the chemoresistance of breast cancer cells to doxorubicin/cyclophosphamide [34]. Given the crucial roles of S100A9 in breast cancer progression and therapeutic resistance, understanding how it is regulated is of utmost importance. Emerging evidence suggests that benzyl butyl phthalate (BBP) induces expression and secretion of S100A9 in tumor microenvironment cells of breast cancer including tumor‑associated dendritic cells and tumor infiltrating myeloid-derived suppressor cells [34]. Additionally, IL-1 receptor-associated kinase 1 (IRAK1) regulates the expression of S100A9 in chromosome 1q21.3-amplified breast cancer cells [28]. However, the regulatory mechanism for S100A9 in TNBC cells is largely unknown.

Our recent quantitative proteomic analysis found that the expression levels of BAP18 were upregulated in TNBC tissues relative to adjacent normal tissues, but its biological function and the underlying molecular mechanism in TNBC progression remain unexplored. In this study, we provide evidence for the first time that BAP18 promotes TNBC progression through, at least in part, transcriptional activation of S100A9. These findings may lead to the development of new targeted therapies to improve clinical outcome of patients with TNBC.

## Materials and methods

### Cell culture and reagents

Human embryonic kidney 293 T (HEK293T) cell line, human mammary epithelial cell line MCF10A, and TNBC cell lines (MDA-MB-231, LM2-4175, MDA-MB-468, MDA-MB-157, BT549, BT-20, SUM149PT, SUM159PT, HCC1806, HCC1937, and Hs578T) were obtained from the Cell Bank of Chinese Academy of Sciences (Shanghai, China) and Shanghai Key Laboratory of Breast Cancer (Fudan University, Shanghai, China). All cell lines were authenticated by short tandem repeat profiling and tested as mycoplasma free. Cells were cultured in DMEM (BasalMedia, #L110) containing 10% fetal bovine serum (FBS; ExCell Biol, #FSP500) and 1 × penicillin- streptomycin (BasalMedia, #S110B). MCF10A cells were cultured in DMEM/F12 (BasalMedia, #L360) containing with 5% donor horse serum (Thermofisher, #16050122), 20 ng/mL epidermal growth factor (EGF; Sino Biological Inc., #10605-HNAE), 10 mg/mL insulin (Yeasen, #40107ES76), 100 ng/mL cholera toxin (Sigma-Aldrich, #C8052), and 0.5 mg/mL hydrocortisone (Yeasen, #40109ES08). Other chemicals and regents were purchased from Sigma-Aldrich unless noted.

### Tissue samples

A total of 10 pairs of primary TNBC tissues and adjacent normal tissues were collected from breast cancer patients who underwent surgery at Fudan University Shanghai Cancer Center. No patients had received chemotherapy, radiotherapy, or targeted therapy before surgery. Adjacent normal breast tissues were histologically confirmed as being free of tumor cells. All procedures were performed following the Ethical Principles for Medical Research Involving Human Subjects. Informed consent was obtained from all subjects.

### DNA constructs, transfection, and viral transduction

To generate Flag-BAP18 or Flag-S100A9 construct, corresponding cDNAs were amplified by PCR and then subcloned into the lentiviral vector pCDH-CMV-MCS-EF1-Puro (System Biosciences, #CD511B-1). The primers used for molecular cloning are provided in Supplementary Tables S1. DNA sequence was verified by DNA sequencing (HuaGene Biotech, Shanghai, China). Transfections were performed using Lipofectamine 2000 (Invitrogen, #11668019) or Neofect DNA transfection reagents (TengyiBio, #TF201201). To generate stable cell lines, expression constructs in lentiviral expression vectors were transfected into HEK293T cells together with packaging plasmid mix. Supernatants were collected after 48 h of transfection and used for infecting cells when cell density reached 70–80% confluence in the presence of 8 mg/mL of polybrene (Sigma-Aldrich, #H9268). After 48 h of infection, cells were selected with 2 mg/mL of puromycin (Sangon Biotech, #A610593) for 1–2 weeks. Short hairpin RNA (shRNA) targeting BAP18 (shBAP18), small interfering RNA (siRNA) targeting S100A9 (siS100A9), and corresponding negative controls were purchased from GenePharma (Shanghai, China). Knockdown efficiency was validated by immunoblotting after 48 h of transfection. The shRNA and siRNA targeting sequences are described in Supplementary Table S2.

### RNA isolation and quantitative real-time PCR

Total RNA was isolated from cultured cells using TRIzol reagent (Thermofisher, #15596018), and cDNA was generated using PrimeScript RT Master Mix (Takara, #RR036A) according to the manufacturer’s protocol. The cDNA product was subjected to quantitative real-time PCR (qPCR) using SYBR Premix Ex Taq (Tli RNaseH Plus) (Takara, #RR420B). Expression levels of target genes were normalized to those of the housekeeping gene GAPDH, and data are present as mean ± SD. qPCR primers were synthesized in HuaGene Biotech (Shanghai, China) and sequences are described in Supplementary Table S3. All assays were repeated at least three times.

### Antibodies and immunoblotting

The detailed information for primary antibodies used in this study is described in Supplementary Table S4. For immunoblotting analyses, cells or tissue samples were lysed in RIPA buffer with 1 × protease inhibitors and phosphatase inhibitors (Bimake, #B14002 and #B15003, respectively). Quantification of protein concentrations was performed using the bicinchoninic acid assay kit (Yeasen, # 20201ES90). Equal amounts of protein extracts were separated by SDS-PAGE and transferred onto PVDF membranes (Millipore, #IPVH00010). The membranes were incubated with indicated primary antibodies and detected with Super ECL detection kit (Yeasen, # 36208ES76). Quantitation of immunoblotting bands was performed using ImageJ software, and expression levels of target proteins were normalized to those of internal control Vinculin.

### Chromatin immunoprecipitation (ChIP)-quantitative PCR (qPCR) assays

ChIP assays were performed using a SimpleChIP Enzymatic Chromatin IP Kit (Magnetic Beads) (Cell Signaling Technology, #9003) according to the manufacturer’s instructions. IgG was used as a negative control, and all data are presented as corresponding fold change relative to Input. The primers used for ChIP-qPCR analysis are listed in Supplementary Table S5.

### Dual-luciferase reporter assays

S100A9 promoter (1000 bp upstream of transcription start site) was cloned into the pLG3-basic vector. Then, pGL3 or pGL3-S100A9 was transfected into the HEK293T cells using Neofect DNA transfection reagents. After 48 h of transfection, the promoter activity of S100A9 was determined using the dual-luciferase reporter assay system (Promega, #E1910) according to the manufacturer’s protocol. All assays were repeated at least three times.

### Cell viability and colony formation assays

A total of 1 × 10^3^ cells were seeded into 6-well plate for colony formation survival assays or 96-well plates for Cell Counting Kit-8 (CCK-8) assays in triplicates. Cell viability was determined using CCK-8 kit (Yeasen, #40203ES92) following the manufacturer’s protocol. Briefly, 10 μl CCK-8 solution and 90 μl medium were added to each well. The plates were incubated in an incubator for 2 h, and then absorbance at 450 nm was measured. For colony formation assays, 1 × 10^3^ cells were cultured in 6-well plates for 12 days, stained with 0.2% crystal violet, and the number of survival colonies was counted.

### Transwell migration and invasion assays

For transwell migration and invasion assays, cells at a density of 4 × 10^4^ per well without FBS were seeded into upper transwell chamber (Corning Falcon, #353097) for cell migration assays and upper Matrigel invasion chamber (Corning BioCoat, #354480) for cell invasion assays. A total of 800 μl culture medium containing 10% FBS was added into the lower chamber. After 16 h of incubation, the migrated and invaded cells were fixed with methanol and then stained with 1% crystal violet. Then, cells on the upper surface were gently removed with a cotton swab. Cells on the lower surface were counted under a light microscope with a magnification of 100. All assays were conducted in triplicate and repeated at least three times.

### Tumor xenografts and lung metastasis in nude mice

The experiments involving animals were performed according to the institutional ethical guidelines. The study was also approved by the Institutional Animal Care and Use Committee of Fudan University. For subcutaneous inoculation, cells were injected into the mammary fat pad of 6-week-old female BALB/c athymic nude mice (*n* = 12; Shanghai SLAC Laboratory Animal Co., Ltd, Shanghai, China). The tumors were measured every three days after tumor formation and the tumor volume was calculated by the formula of (length × width^2^)/2. Mice were killed at the indicated times in figure legends. The removed tumors were weighed. For experimental lung metastasis assays, 1.5 × 10^6^ LM2-4175 cells stably expressing shNC or shBAP18 were injected into the tail vein of 6-week-old female BALB/c athymic nude mice (*n* = 6; Shanghai SLAC Laboratory Animal Co., Ltd, Shanghai, China). Mice were sacrificed after the inoculation for 8 weeks. The lungs from mice were removed and metastatic nodules on them were counted. For all animal experiments, mice were randomly divided into different groups, and the investigators were not blinded to the group allocation. No statistical methods were used to estimate the number of mice.

### Statistical analysis

All experiments were replicated at least three times and the results are presented as mean ± standard error. The unpaired two tailed Student’s *t*-test was used to compare the difference between two groups. All statistical analyses were conducted with SPSS version 22.0 software. A *p*-value less than 0.05 was considered statistically significant.

## Results

### Expression levels of BAP18 are upregulated in TNBC tissues

**Fig. 1:**
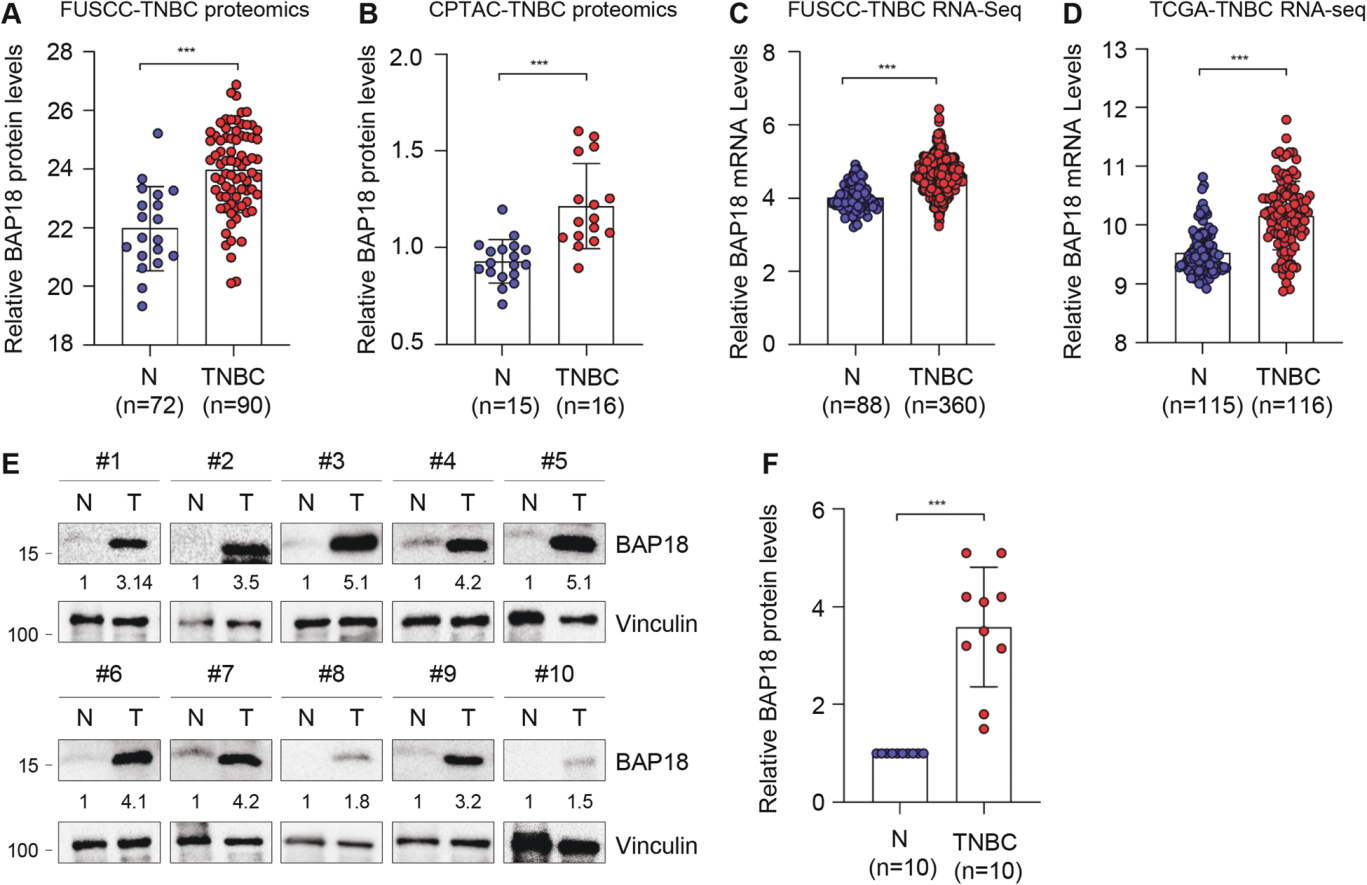
Expression levels of BAP18 are upregulated in TNBC tissues. **A** Relative BAP18 protein levels in FUSCC-TNBC proteomic assays. FUSCC, Fudan University Shanghai Cancer Center. N, Normal tissues. **B** Relative BAP18 protein levels in CPTAC database. **C** Relative BAP18 mRNA expression levels in FUSCC-TNBC RNA-seq dataset [35]. **D** Relative BAP18 mRNA Levels in TCGA RNA-seq database. **E**, **F** Cellular lysates from 10 pairs of primary TNBC specimens and matched adjacent normal tissues were subjected to immunoblotting analysis with an anti-BAP18 antibody. Representative immunoblotting images are shown in **E** and corresponding quantitative results are presented in **F**. Quantitation of immunoblotting bands was performed using Image J software, and expression levels of BAP18 were normalized to those of Vinculin. ***p < 0.001.

To gain insights into the molecular mechanisms of TNBC progression, we recently carried out quantitative proteomic analysis using 90 cases of TNBC tissues and 72 cases of matched adjacent normal tissues, and found that the expression levels of BAP18 were upregulated in TNBC tissues relative to normal tissues (Fig. 1A). This result was further confirmed in the Clinical Proteomic Tumor Analysis Consortium (CPTAC) database (https://cptac-data-portal.georgetown.edu/), which includes mass spectrometry-based global proteomics data for 16 TNBC specimens and 15 normal breast samples (Fig. 1B). Moreover, analysis of our recently published RNA-seq dataset of TNBC, which includes the mRNA profiles of 360 cases of TNBC tissues and 88 cases of adjacent normal tissues [35], revealed that mRNA levels of BAP18 were increased in TNBC tissues (Fig. 1C). Similar result was also obtained in the RNA-seq dataset of TNBC from The Cancer Genome Atlas (TCGA) (Fig. 1D). To further validate these results, we collected 10 pairs of primary TNBC tumor specimens and matched adjacent normal tissues to detect the protein levels of BAP18 by immunoblotting. As shown in Fig. 1E, F, BAP18 was overexpressed in TNBC samples as compared with normal tissues. Collectively, these results suggest that BAP18 is upregulated in TNBC tissues.

### BAP18 promotes TNBC cell proliferation, migration, and invasion in vitro

**Fig. 2:**
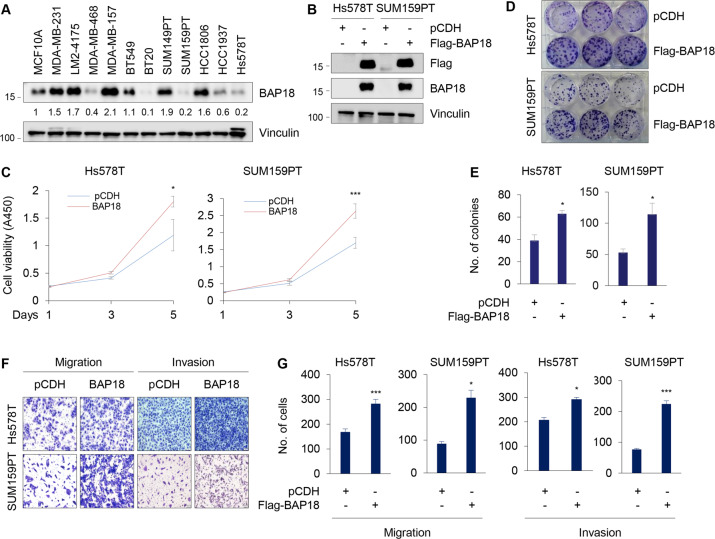
Ectopic expression of BAP18 promotes TNBC cell proliferation, migration, and invasion in vitro. **A** Cellular lysates from 11 TNBC cell lines and human mammary epithelial cell line MCF10A were subjected to immunoblotting analysis with the indicated antibodies. **B** Ectopic expression of BAP18 in SUM159PT and Hs578T cells by lentiviral infections. The expression status of BAP18 in these cell lines was validated by immunoblotting. **C**–**E** SUM159PT and Hs578T cells stably expressing pCDH and Flag-BAP18 were subjected to CCK-8 assays (**C**) and colony formation assays (**D**–**E**). Representative images of survival colonies and corresponding quantitative results are shown in **D** and **E**, respectively. **F**, **G** SUM159PT and Hs578T cells stably expressing pCDH and Flag-BAP18 were subjected to Transwell migration and Matrigel invasion assays. Representative images of migrated and invaded cells and corresponding quantitative results are shown in **F** and **G**, respectively. *p < 0.05; ***p < 0.001.

To address the biological functions of BAP18 in TNBC progression, we first examined the expression levels of BAP18 in 11 commonly used TNBC cell lines and human mammary epithelial cell line MCF10A. As shown in Fig. 2A, MDA-MB-231, LM2-4175, MDA-MB-157, SUM149PT, and HCC1806 cell lines expressed relatively higher levels of BAP18 than MDA-MB-468, BT20, SUM159PT, HCC1937, and Hs578T cell lines did. We next stably expressed BAP18 in Hs578T and SUM159PT cells by lentiviral infections. The expression status of BAP18 in these established stable cell lines was validated by immunoblotting (Fig. 2B). CCK-8 and colony formation assays showed that ectopic expression of BAP18 in Hs578T and SUM159PT cells accelerated cell proliferation (Fig. 2C) and colony formation (Fig. 2D, E) compared to empty vector control. As TNBC is the most aggressive type of breast cancer with highly invasive and metastatic phenotype [6], we next investigated the impact of BAP18 on migratory and invasive potential of TNBC cells. Transwell migration and Matrigel invasion assays showed that Hs578T and SUM159PT cells stably expressing BAP18 displayed increased migratory and invasive potential compared to empty vector expressing control cells (Fig. 2F, G).

**Fig. 3:**
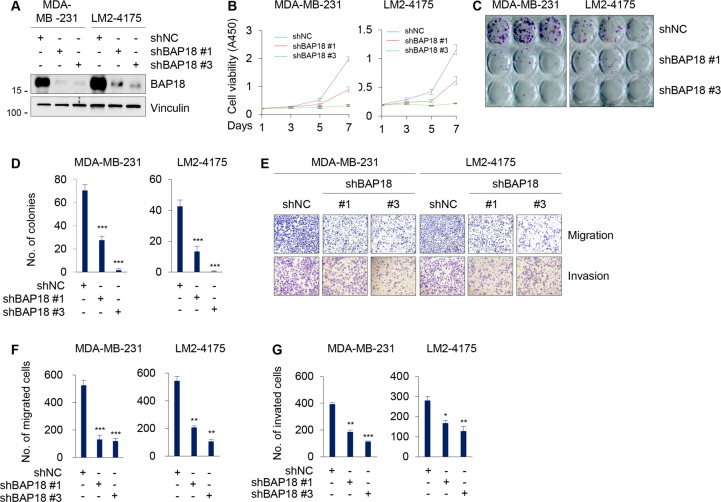
Depletion of endogenous BAP18 attenuates TNBC cell proliferative, migratory, and invasive potential in vitro. **A** Knockdown of endogenous BAP18 in MDA-MB-231 and LM2-4175 cells by lentiviral infections with two independent shRNAs targeting BAP18 (shBAP18 #1 and #3). The expression status of BAP18 in these cell lines was validated by immunoblotting. **B**–**D** MDA-MB-231 and LM2-4175 cells stably expressing shNC and shBAP18 were subjected to CCK-8 assays (**B**) and colony formation assays (**C**, **D**). Representative images of survival colonies and corresponding quantitative results are shown in **C** and **D**, respectively. **E**–**G** MDA-MB-231 and LM2-4175 cells stably expressing shNC and shBAP18 were subjected to Transwell migration and Matrigel invasion assays. Representative images of migrated and invaded cells (**E**) and corresponding quantitative results (**F**, **G**) are shown, respectively. *p < 0.05; **p < 0.01; ***p < 0.001.

To further validate the above results, we then knocked down endogenous BAP18 in MDA-MB-231 and LM2-4175 cell lines using two independent shBAP18 (#1 and #3). The efficiency of shRNA-mediated BAP18 knockdown in these established stable cell lines was confirmed by immunoblotting (Fig. 3A). CCK-8 and colony formation assays showed that knockdown of endogenous BAP18 in MDA-MB-231 and LM2-4175 cells reduced cell proliferation (Fig. 3B) and colony formation potential (Fig. 3C, D). Transwell migration and invasion assays revealed that knockdown of BAP18 attenuated migratory and invasive potential of MDA-MB- 231 and LM2-4175 cells (Fig. 3E–G). Taken together, these results suggest that BAP18 promotes TNBC cell proliferative, migratory, and invasive potential in vitro.

### BAP18 promotes xenograft tumor growth and lung metastasis of TNBC cells in vivo

**Fig. 4:**
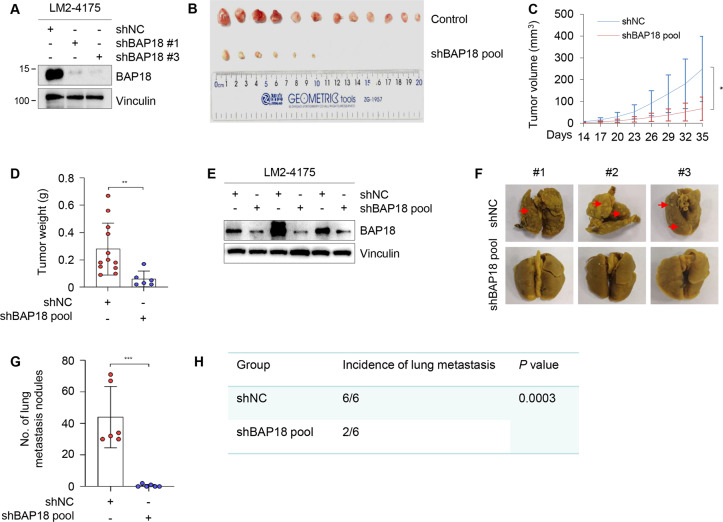
Knockdown of BAP18 impairs xenograft tumor growth and lung metastasis of TNBC cells in vivo. **A** The efficiency of BAP18 knockdown in LM2-4175 cells was verified by immunoblotting. **B**–**D** LM2-4175 cells (1 × 10^6^) stably expressing shNC or shBAP18 pool (#1 and #3) were subcutaneously injected into mammary fat pads of 6-week-old female BALB/c nude mice (*n* = 12). Five weeks after injection, xenograft tumors were removed from nude mice and photographed (**B**). The volume and weight of xenograft tumors are shown in **C** and **D**, respectively. **E** The expression status of BAP18 in xenograft tumor tissues was verified by immunoblotting. **F**–**H** LM2-4175 cells (1.5 × 10^6^) stably expressing shNC or shBAP18 pool (#1 and #3) were injected into nude mice through tail veins, and the number of metastatic lung nodules was counted after 8 weeks of injection. The representative images of metastatic lung tumors (**F**), the number of metastatic lung nodules (**G**), and incidence of lung metastasis (**H**) in nude mice are shown. *p < 0.05; **p < 0.01; ***p < 0.001.

To examine whether BAP18 affects tumorigenic capacity of TNBC cells in vivo, LM2-4175 cells stably expressing shNC or shBAP18 pool (#1 and #3) (Fig. 4A) were subcutaneously injected into mammary fat pads of 6-week-old female BALB/c nude mice. Five weeks after injection, xenograft tumors were removed from nude mice, photographed, and weighted (Fig. 4B). In support of in vitro findings, the volume and weight of xenograft tumors from LM2-4175 cells stably expressing shBAP18 pool were much lower than those of xenograft tumors from cells expressing shNC (Fig. 4C, D). Immunoblotting analysis also demonstrated that the protein expression levels of BAP18 were lower in xenograft tumor tissues expressing shBAP18 than those in shNC expressing tumor tissues (Fig. 4E).

To examine whether BAP18 affects the metastatic potential of TNBC cells in vivo, LM2-4175 cells stably expressing shNC or shBAP18 pool (#1 and #3) were injected into nude mice through tail veins, and the number of metastatic lung nodules was counted after 8 weeks of injection. Results showed that knockdown of BAP18 reduced the number of metastatic lung tumors (Fig. 4F, G) and incidence of lung metastasis (Fig. 4H) in nude mice compared to control group. Collectively, these results suggest that BAP18 functions as an emerging oncoprotein in TNBC progression in vivo.

### S100A9 is a downstream target gene of BAP18

**Fig. 5:**
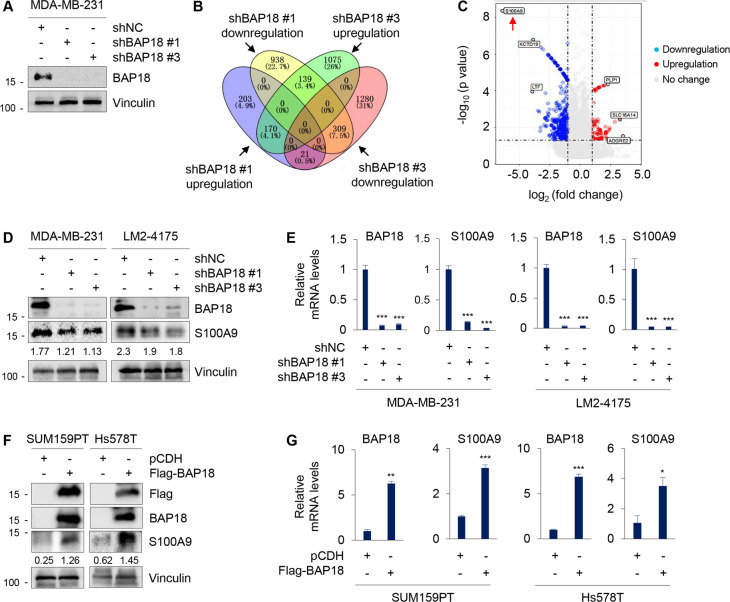
S100A9 is a downstream target gene of BAP18. **A** shRNA-mediated knockdown of BAP18 in MDA-MB-231 cells was verified by immunoblotting with the indicated antibodies. **B** Differentially expressed genes between shBAP18 (#1 and #3) and shNC group according to fold change ≥ 2 or ≤ −2. **C** Volcanic map of differentially expressed genes. **D**, **E** The protein (**D**) and mRNA (**E**) levels of S100A9 in MDA-MB-231 and LM2-4175 cells stably expressing shNC and shBAP18. **F**, **G** The protein (**F**) and mRNA (**G**) levels of S100A9 in SUM159PT and Hs578T cells stably expressing pCDH and Flag-BAP18. *p < 0.05; **p < 0.01; ***p < 0.001.

To investigate the molecular mechanisms by which BAP18 promotes TNBC progression, MDA-MB-231 cells stably expressing shNC and two shBAP18 (#1 and #3) were subjected to RNA-Seq analysis to identify downstream target genes of BAP18 (Fig. 5A). According to the threshold (fold change ≥2 or ≤ −2) of differential gene expression, 170 genes were upregulated and 309 genes were downregulated in cells expressing shBAP18 (#1 and #3) as compared with shNC expressing cells (Fig. 5B). Among them, the most significantly downregulated gene in cells expressing shBAP18 was S100A9 (Fig. 5C). Immunoblotting and qPCR analyses demonstrated that the protein and mRNA levels of BAP18 were decreased in BAP18-depleted MDA-MB-231 and LM2-4175 cells (Fig. 5D, E), but were increased in BAP18-overexpressing SUM159PT and Hs578T cells (Fig. 5F, G) as compared with corresponding control cells. Analysis of our recently published TNBC RNA-Seq dataset [35] revealed that the mRNA levels of BAP18 were positively correlated with those of S100A9 (Supplementary Fig. S1). These results suggest that BAP18 positively regulates S100A9 transcription in TNBC cells.

**Fig. 6:**
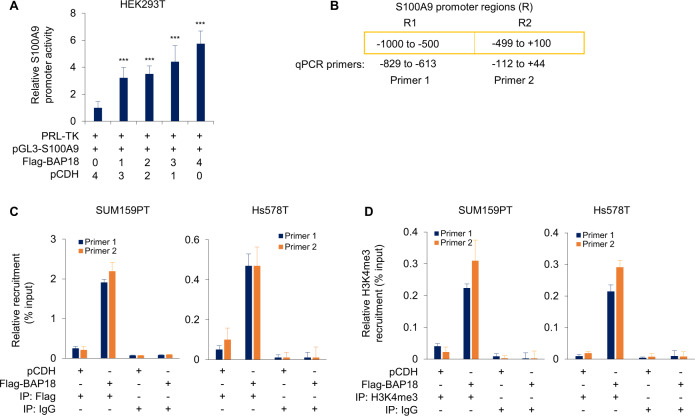
BAP18 is recruited to S100A9 promoter and enhances its promoter activity. **A** HEK293T cells were transfected with the pGL3-S100A9 luciferase reporter plasmid alone or in combination with increasing doses of Flag-BAP18. pRL-TK reporter plasmid was also transfected into HEK293T cells as an internal control for transfection efficiency. After 48 h of transfection, cells were lysed and luciferase activities in triplicate samples were determined. Luciferase values (relative light units) were calculated and normalized to controls. Values are reported as the fold-increase and represent the mean ± SD from three independent transfection experiments. **B** Line diagram showing the regions of S100A9 promoter analyzed. **C** ChIP analysis for the recruitment of BAP18 onto the S100A9 promoter in SUM159PT and Hs578T cells stably expressing pCDH and Flag-BAP18. **D** ChIP analysis for the recruitment of H3K4me3 onto the S100A9 promoter in SUM159PT and Hs578T cells stably expressing pCDH and Flag-BAP18. ***p < 0.001.

To examine whether BAP18 affects the promoter activities of S100A9, we transfected HEK293T cells with a S100A9 promoter reporter plasmid alone or in combination with increasing dose of Flag-BAP18, and determined S100A9 luciferase activities after 48 h of transfection. As shown in Fig. 6A, BAP18 enhanced S100A9 promoter activities in a dose-dependent manner. To examine the possibility of the recruitment of BAP18 onto the S100A9 promoter, we then designed two pairs of qPCR primers to amplify promoter region (−1000 to +100) of S100A9 following chromatin immunoprecipitation (ChIP) assays (Fig. 6B). ChIP-qPCR assays showed that BAP18 was recruited onto two regions (−829 to −613 and −112 to +44) of the S100A9 promoter (Fig. 6C). As BAP18 is a reader of histone marker H3K4me3 [20], we next examined the enrichment of H3K4me3 onto S100A9 promoter regions by ChIP-qPCR assays using the same qPCR primers used for BAP18 ChIP-qPCR assays. Results showed that H3K4me3 was enriched at the same regions of S100A9 promoter, where BAP18 was occupied (Fig. 6D). These results collectively demonstrated that S100A9 is a downstream target gene of BAP18.

### BAP18 promotes TNBC tumor growth partially through transcriptional transaction of S100A9

To validate previous findings that S100A9 promotes breast cancer progression, we first knocked down S100A9 in MDA0MB-231 cells using two independent siRNAs targeting S100A9 (siS100A9 #1 and #2) (Supplementary Fig. S2A). CCK-8, Transwell migration and invasion assays showed that depletion of S100A9 attenuated the proliferative, migratory, and invasive potential of MDA-MB-231 cells (Supplementary Fig. S2B−S2D).

**Fig. 7:**
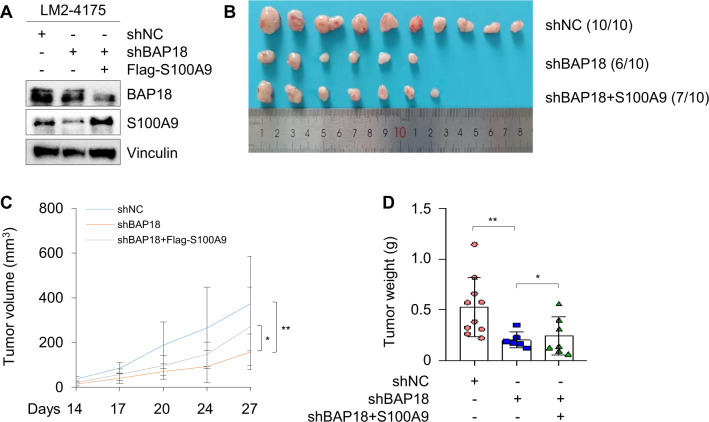
BAP18 promotes TNBC tumor growth partially through transcriptional transaction of S100A9. **A** Validation of BAP18 and S100A9 in LM2-4175 cells stably expressing shNC, shBAP18 alone or in combination with Flag-S100A9 by immunoblotting. **B**−**D** LM2-4175 cells (1 × 10^6^) stably expressing shNC, shBAP18 alone or in combination with Flag-S100A9 were subcutaneously injected into the mammary fat pads of 6-week-old female BALB/c nude mice (*n* = 10). After 4 weeks of injection, xenograft tumors were removed from nude mice and photographed (**B**). Tumor volume and weight of xenograft tumors are shown in **C** and **D**, respectively. *p < 0.05; **p < 0.01.

To address whether BAP18 promotes TNBC tumor growth through regulating S100A9, we re-expressed Flag-S100A9 in LM2-4175 cells stably expression shBAP18 (Fig. 7A), and then 1 × 10^6^ cells were injected into the mammary fat pad of 6-week-old female BALB/c athymic nude mice. Results showed that knockdown of BAP18 in LM2-4175 cells decreased xenograft tumor incidence, volume, and weight, and the noted effects were partially reverted by reintroduction of Flag-S100A9 into LM2-4175 cells expressing shBAP18 (Fig. 7B−D, respectively). These results demonstrated that BAP18 promotes TNBC tumor growth through, at least in part, transcriptional transaction of S100A9.

## Discussion

Dysregulation of epigenetic regulators drives aberrant transcriptional programs that promote tumor progression and therapeutic resistance [36]. A case in point is bromodomain-containing protein 4 (BRD4), which is a chromatin reader protein critical for TNBC cell migration, invasion, and metastasis through regulating Jagged1 expression [9]. Pharmacological inhibition of BRD4 using BET bromodomain inhibitors results in blocking TNBC growth and progression [10, 11]. In this study, we provide the first evidence that BAP18, a reader for histone mark H3K4me3, functions as an oncoprotein in TNBC progression.

BAP18 is a poorly characterized protein, particularly its functional and mechanistic roles in human cancer remains largely elusive. Very limited information shows that BAP18 promotes cell growth of prostate tumor [19] and oral squamous cell carcinoma [21]. In addition, expression levels of BAP18 are associated with cellular sensitivity of hepatocellular carcinoma to sorafenib [37] and of ERα-positive breast cancer to antiestrogen therapy [20]. Our quantitative proteomic assays, immunoblotting analysis of clinical TNBC specimens, and analysis of publicly available database demonstrated that the protein and mRNA levels of BAP18 are upregulated in a subset of primary TNBC tumors (Fig. 1). Gain- and loss-of-function assays showed that BAP18 promotes TNBC cell proliferation, migration, and invasion in vitro and xenograft tumor growth and lung colonization in vivo (Figs. 2–4). These results suggest that BAP18 is an emerging oncoprotein in TNBC.

S100A family of proteins have been shown to regulate multiple biological functions related to cancer progression, metastasis, immunosuppression, and therapeutic resistance [22]. Previous studies demonstrated that S100A9 is frequently upregulated in breast tumors [23–25], and its overexpression is closely associated with metastatic progression [24, 26–29], chemoresistance [34], and poor prognosis of patients with breast cancer [25, 30, 31]. Despite its functional importance in breast cancer progression, the regulatory mechanism for S100A9 in TNBC cells is largely unknown. In this study, we showed that S100A9 is a transcriptional target of BAP18 (Figs. 5 and 6). BAP18 was recruited to H3K4me3-marked promoter of S100A9, and enhanced its promoter activities (Fig. 6). In agreement with previous studies, we demonstrated that depletion of S100A9 suppressed TNBC cell proliferation, migration, and invasion (Supplementary Fig. S2). Moreover, re-expression of S100A9 in BAP18-depleted TNBC cells partially rescued reduced xenograft tumor growth in mice by BAP18 knockdown (Fig. 7).

Taken together, findings presented here suggest that BAP18 promotes TNBC progression through, at least in part, transcriptional regulation of S100A9 and may represent a potential therapeutic target for TNBC.

## Supplementary information

Supplementary information

Supplementary Material (original WB data)

Reproducibility checklist

## Data Availability

All data generated or analyzed during this study is included in this paper, and is available on request from the corresponding author.
