# Supplementary material for: Chromatin complexes subunit BAP18 promotes triple-negative breast cancer progression through transcriptional activation of oncogene S100A9
Source: Cell Death Dis. 2022 Apr 28;13(4):408. doi: 10.1038/s41419-022-04785-x (PMC9050672; doi:10.1038/s41419-022-04785-x)
Supplement: Supplementary file 1 — Supplementary information [file 41419_2022_4785_MOESM1_ESM.docx]

**Supporting information for**

Zhang YL, et al. Chromatin complexes subunit BAP18 promotes triple-negative breast cancer progression through transcriptional activation of oncogene S100A9

Supplementary information includes:

Supplementary Figures: 2

Supplementary Tables: 5

**Supplementary Figure and Figure legends**

FUSCC-TNBC RNA-Seq

**
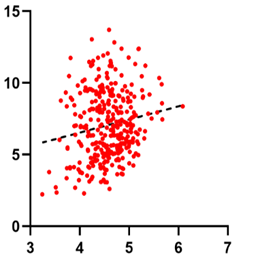
**

S100A9 mRNA levels

BAP18 mRNA levels

**Figure S1.** The correlation of the expression levels between BAP18 and S100A9 in FUSCC-TNBC RNA-Seq dataset.

Analysis of the correlation of the expression levels between BAP18 and S100A9 in our recently published TNBC RNA-Seq dataset, which includes the mRNA profiles of 360 cases of TNBC tissues ^1^.The mRNA levels of BAP18 were positively correlated with those of S100A9 (*p*=0.002, R=0.21).


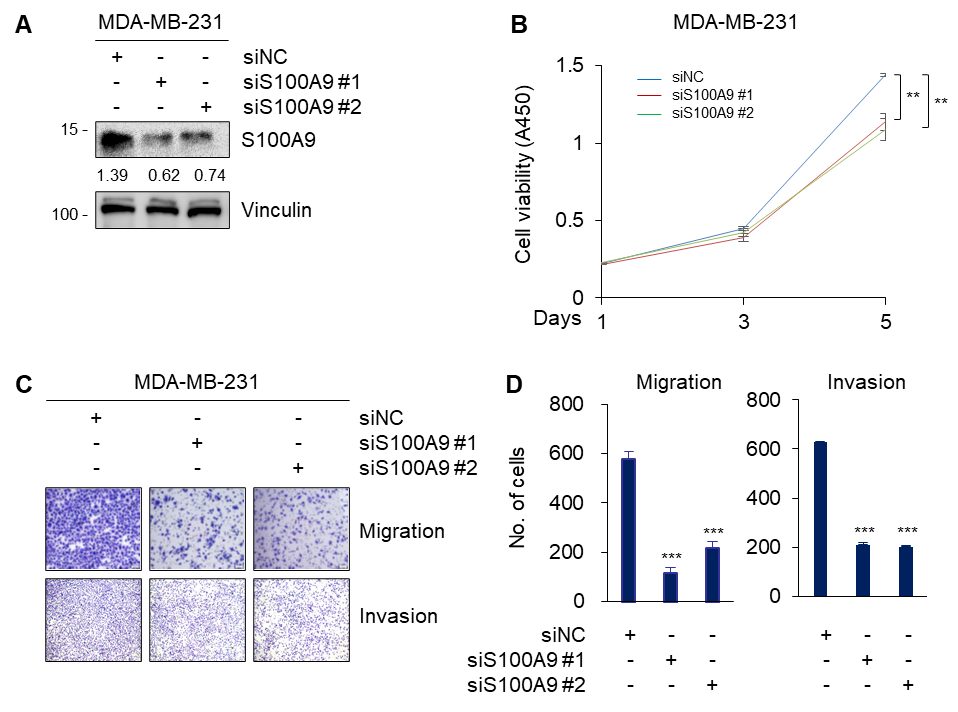


**Figure S2. Knockdown of S100A9 attenuates the proliferative, migratory, and invasive potential of MDA-MB-231 cells**

(**A**) Knockdown of endogenous S100A9 in MDA-MB-231 cells using two independent siRNAs targeting S100A9 (siS100A9 #1 and #2). siRNA-mediated knockdown of S100A9 was verified by immunoblotting. (**B**) MDA-MB-231 cells transfected with siNC and siS100A9 were subjected to CCK-8 assays. (**C-D**) MDA-MB-231 cells transfected with siNC and siS100A9 were subjected to Transwell migration and Matrigel invasion assays. Representative images of migrated and invaded cells (C) and corresponding quantitative results (D) are shown, respectively. **, *p*<0.01; ***, *p*<0.001.

**Supplementary Tables**

**Table S1. Primers used for molecular cloning of Flag-BAP18 expression vectors**

| Plasmid | Primers | Sequence |
| --- | --- | --- |
| Flag-BAP18 | Forward | ATGACGTCAGCGTCCACAAAGGTC |
|  | Reverse | GGCCTGGTCGAAGTTGAGTTTCTTAGC |
| Flag-S100A9 | Forward | ATGACTTGCAAAATGTCGCAGC |
|  | Reverse | GGGGGTGCCCTCCCC |

**Table S2. shRNA and siRNA target sequences**

|  | Sequences |
| --- | --- |
| shBAP18 #1 | CTCTGACCTCTCACTGTTCAT |
| shBAP18 #3 | GAGAAACTCCTCCAGCTAAGA |
| siS100A9 #1 | GCUUCGAGGAGUUCAUCAUTT |
|  | AUGAUGAACUCCUCGAAGCTT |
| siS100A9 #2 | GCAACAUAGAGACCAUCAUTT |
|  | AUGAUGGUCUCUAUGUUGCTT |

**Table S3. Primers for qPCR analysis**

| Genes | Primers | Sequences |
| --- | --- | --- |
| BAP18 | Forward | TGGCATCTGGTGTCTTGTCA |
|  | Reverse | TTGGCATCGGAGTCGTTCA |
| S100A9 | Forward | GCACCCAGACACCCTGAACCA |
|  | Reverse | GTGTCCAGGTCCTCCATGATG |
| GAPDH | Forward | GAAGGTGAAGGTCGGACTC |
|  | Reverse | GAAGATGGTGATGGGATTTC |

**Table S4. Antibodies used in this study**

| Antibodies | Vendors | Cat# | Hosts | Working concentration |
| --- | --- | --- | --- | --- |
| BAP18 | Proteintech | 27114-1-AP | Rabbit | 1:1500 (WB) |
| S100A9 | Abcam | ab92507 | Rabbit | 1:1000 (WB) |
| Vinculin | Sigma | V9131 | Mouse | 1:5000 (WB) |

**Table S5. Primers for ChIP -qPCR analysis**

| Regions | Primers | Sequences |
| --- | --- | --- |
| Region 1 (primer 1) | Forward | AACTGGAGGTTCGTAGCAGG |
|  | Reverse | TCTGGTTCCTGGTTAAGGGC |
| Region 2 (primer 2) | Forward | GCAGCATTACCACACTGCTC |
|  | Reverse | GCTGGCAGCTCACTTACCAA |
