# Supplementary material for: Chromatin complexes subunit BAP18 promotes triple-negative breast cancer progression through transcriptional activation of oncogene S100A9
Source: Cell Death Dis. 2022 Apr 28;13(4):408. doi: 10.1038/s41419-022-04785-x (PMC9050672; doi:10.1038/s41419-022-04785-x)

Figure 1E

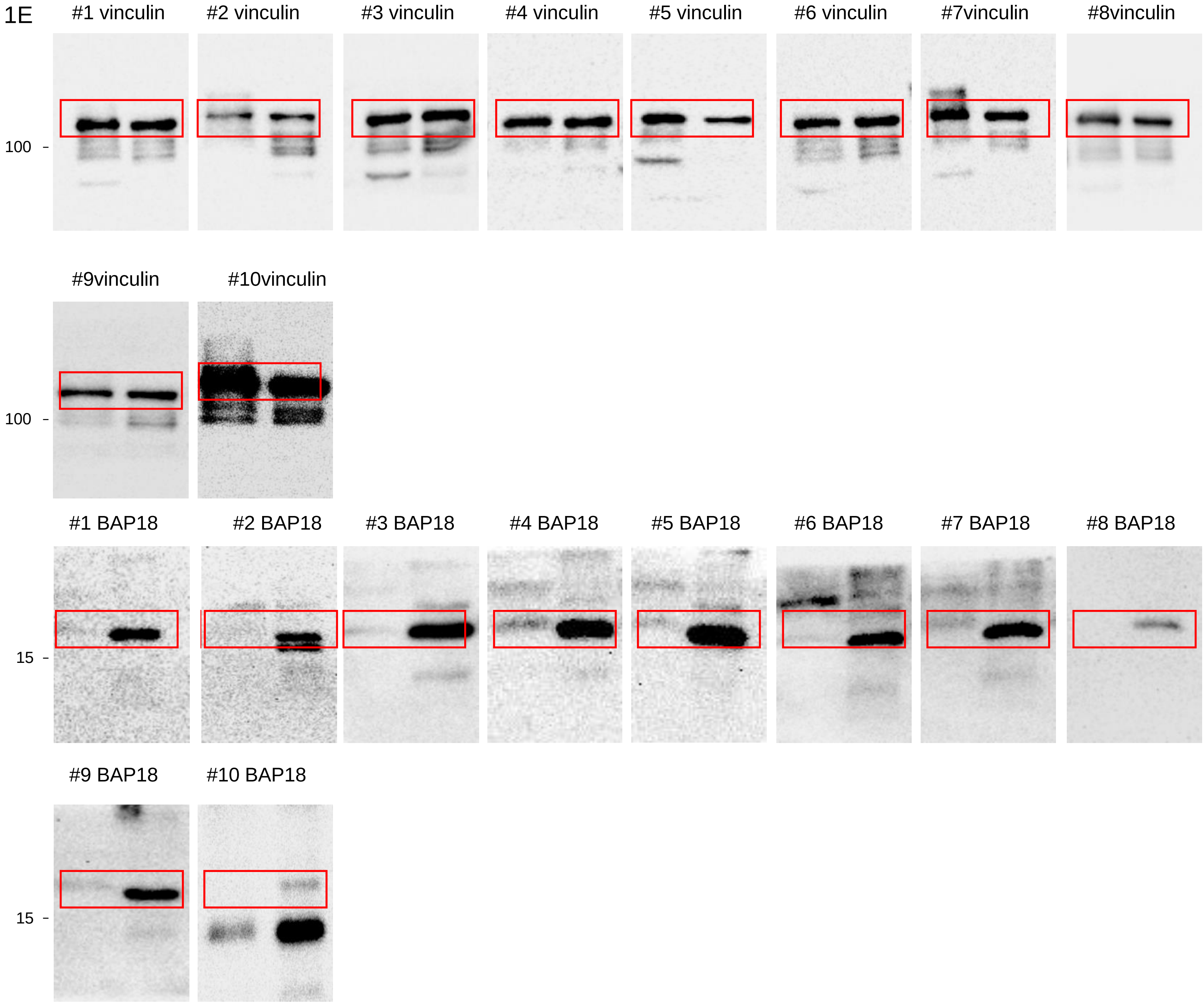
#1 vinculin
#2 vinculin
#3 vinculin
#4 vinculin
#5 vinculin
#6 vinculin
#7vinculin
#8vinculin
100 -
#9vinculin
#10vinculin
100 -
#1 BAP18
#2 BAP18
#3 BAP18
#4 BAP18
#5 BAP18
#6 BAP18
#7 BAP18
#8 BAP18
15 -
#9 BAP18
#10 BAP18
15 -

Figure 2A

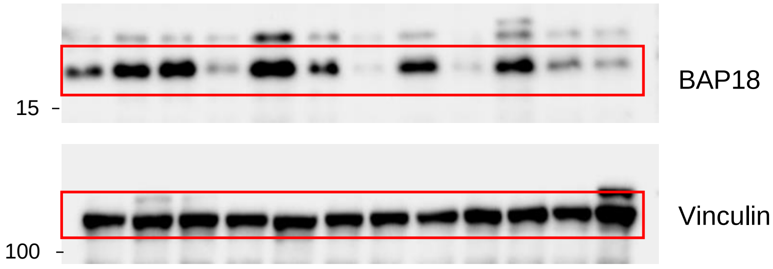

Figure 2B

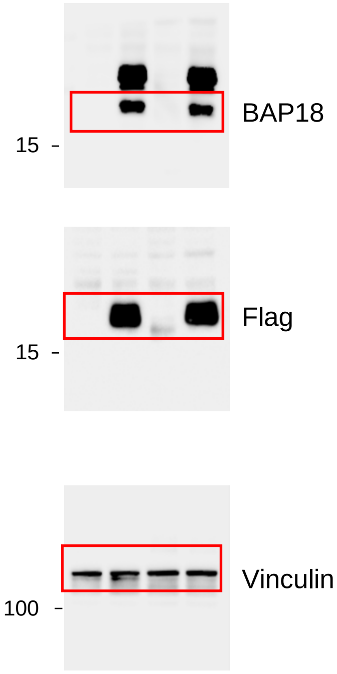

Figure 3A

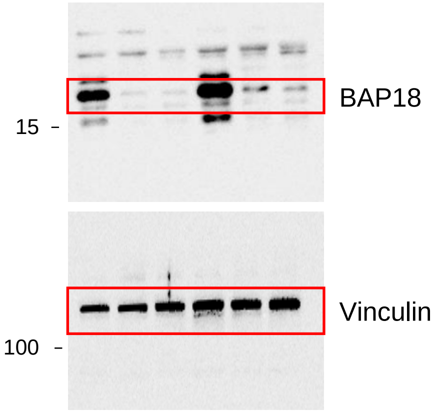

Figure 4E

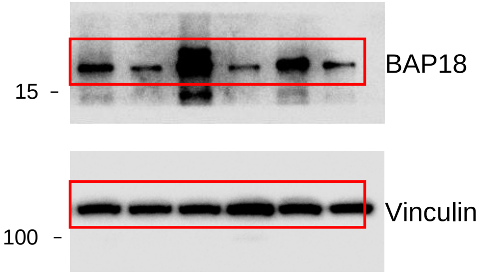

Figure 4A

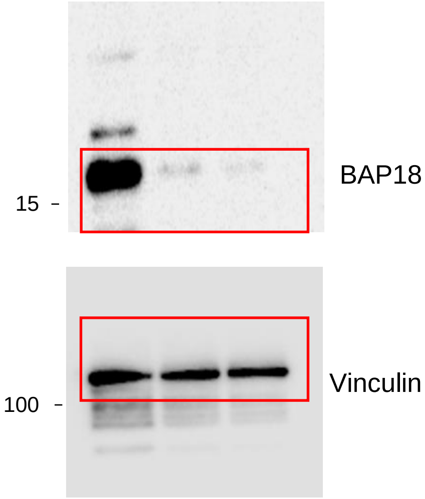

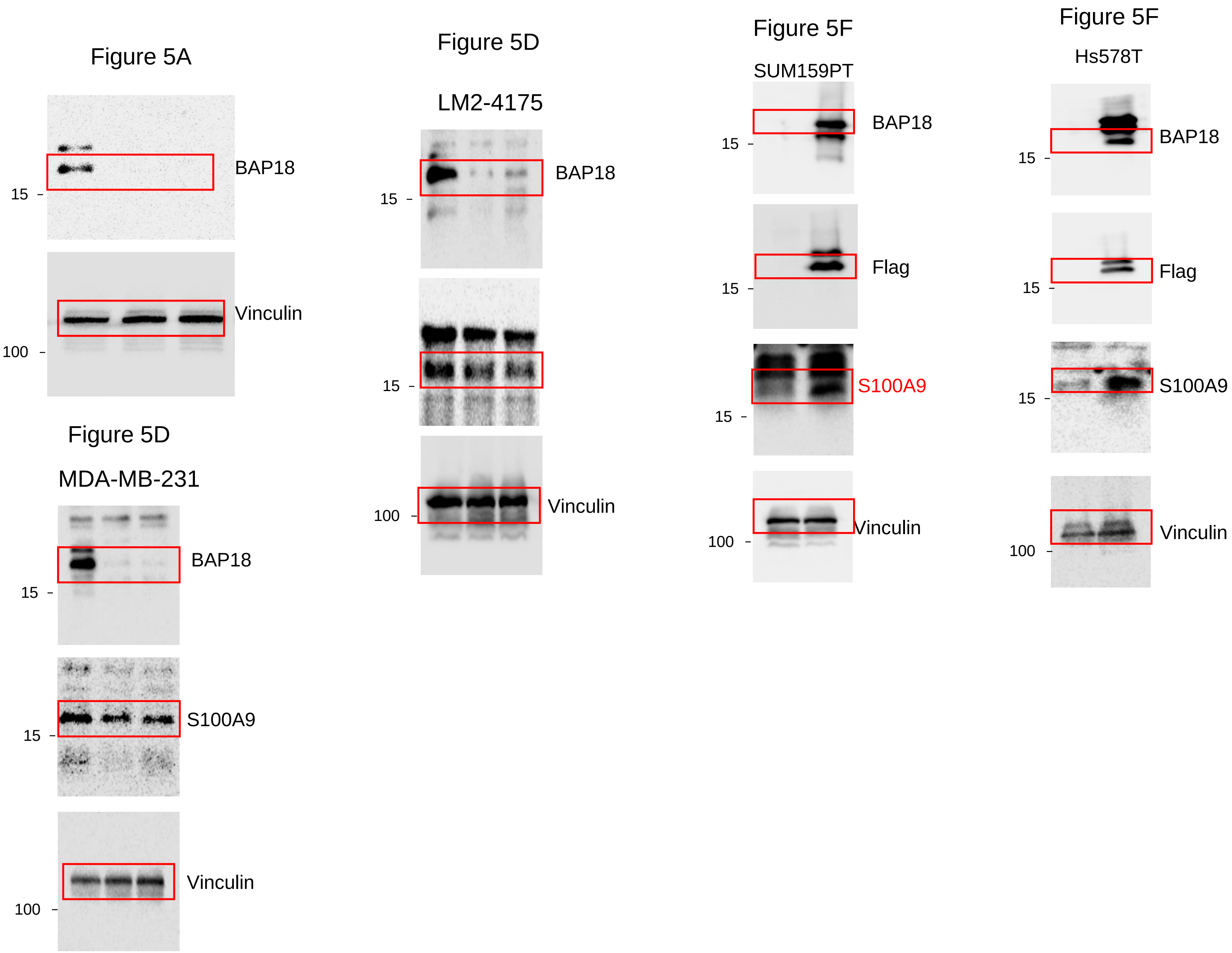

Figure 5A
BAP18
15
Vinculin
100
Figure 5D
MDA-MB-231
BAP18
15
S100A9
15
Vinculin
100
Figure 5D
LM2-4175
BAP18
15
15
Vinculin
100
Figure 5F
SUM159PT
BAP18
15
Flag
15
S100A9
15
Vinculin
100
Figure 5F
Hs578T
BAP18
15
Flag
15
S100A9
15
Vinculin
100

Figure 7A

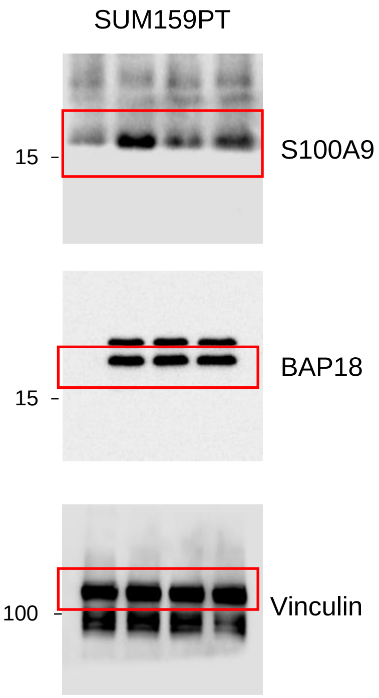

Figure 7A

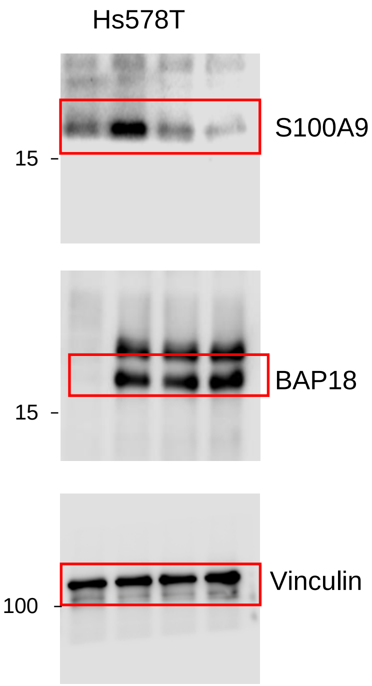

Figure 7C

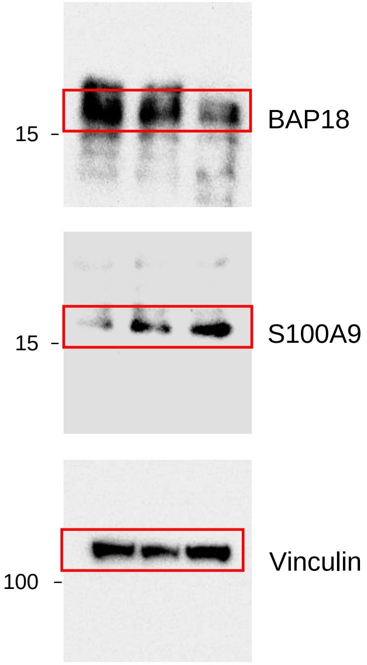

Figure S2-A

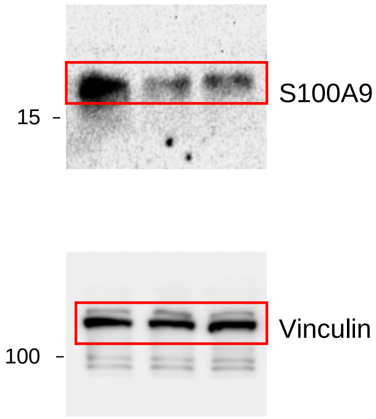

Supplement: Supplementary file 2 — Supplementary Material (original WB data) [file 41419_2022_4785_MOESM2_ESM.pdf]
